# Supplementary material for: Three Birds, One Excipient: Development of an Improved pH, Isotonic, and Buffered Ketamine Formulation for Subcutaneous Injection
Source: Pharmaceutics. 2022 Mar 3;14(3):556. doi: 10.3390/pharmaceutics14030556 (PMC8955626; doi:10.3390/pharmaceutics14030556)
Supplement: Supplementary file 1 [file pharmaceutics-14-00556-s001.zip › pharmaceutics-1553537-supplementary.pdf]

pharmaceutics

Supplementary Materials: Three Birds, One Excipient: Development of an Improved pH, Isotonic, and Buffered Ketamine Formulation for Subcutaneous Injection

Jason Wallach*, James Gamrat, Rebekah Jauhola-Straight, Jeffrey Becker and Thomas Eckrich

Table S1. Results from Fehling's test for stability of Captisol®: Glucose sensitivity test. N = 3.

Sample	Result	Observation (20 minutes post-Fehling test)
Glucose (10 mg/mL)	Positive	Yellow solution, red precipitate
Glucose (5 mg/mL)	Positive	~Colorless solution, red precipitate
Glucose (2 mg/mL)	Positive	Mostly blue solution, red precipitate
Glucose (1 mg/mL)	Inconclusive	Blue solution, inconsistently produced a red precipitate
Glucose (0.75 mg/mL)	Inconclusive	Blue solution, inconsistently produced a red precipitate
Glucose (0.5 mg/mL)	Negative	Blue solution unchanged
40% Captisol® (Negative control)	Negative	Blue solution unchanged
HPLC H_2O (Negative Control)	Negative	Blue solution unchanged

Table S2. Results from Fehling's test for stability of Captisol®: CapAcid prepared from 15% Captisol®. N = 3. Results were evaluated at 20 minutes after Fehling's test. CapAcid concentration from the column solution was determined to be 11.4 ± 0.2 % off the column (mean ± SEM, N = 3), several dilutions of the CapAcid solution were also prepared.

Sample	Result 0 hr	Result 24 hr	Result 48 hr	Result 72 hr
10 mg/mL Glucose (Positive control)	Positive	Positive	Positive	Positive
2% Glucose (Positive control)	Positive	Positive	Positive	Positive
40% Captisol® (Negative control)	Negative	Negative	Negative	Negative
HPLC H_2O (Negative control)	Negative	Negative	Negative	Negative
CapAcid (11.4%)	Negative	Negative	Negative	Negative
CapAcid	Negative	Negative	Negative	Negative

(5.7%)				
CapAcid (2.85%)	Negative	Negative	Negative	Negative

Table S3. Results from Fehling test for stability of Captisol®: CapAcid prepared from 20% Captisol®. N = 3. Results were 20 minutes after Fehling test. Mean concentration of CapAcid solution from the column was determined to be 15.4 ± 0.08 % (mean ± SEM, N = 3).

**Sample**	**Result 0 hr**	**Result 24 hr**	**Result 48 hr**	**Result 168 hr**
10 mg/mL Glucose (Positive control)	Positive	Positive	Positive	Positive
2% Glucose (Positive control)	Positive	Positive	Positive	Positive
40% Captisol® (Negative control)	Negative	Negative	Negative	Negative
HPLC H_2O (Negative control)	Negative	Negative	Negative	Negative
CapAcid (15.4%)	Negative	Negative	Negative	Positive

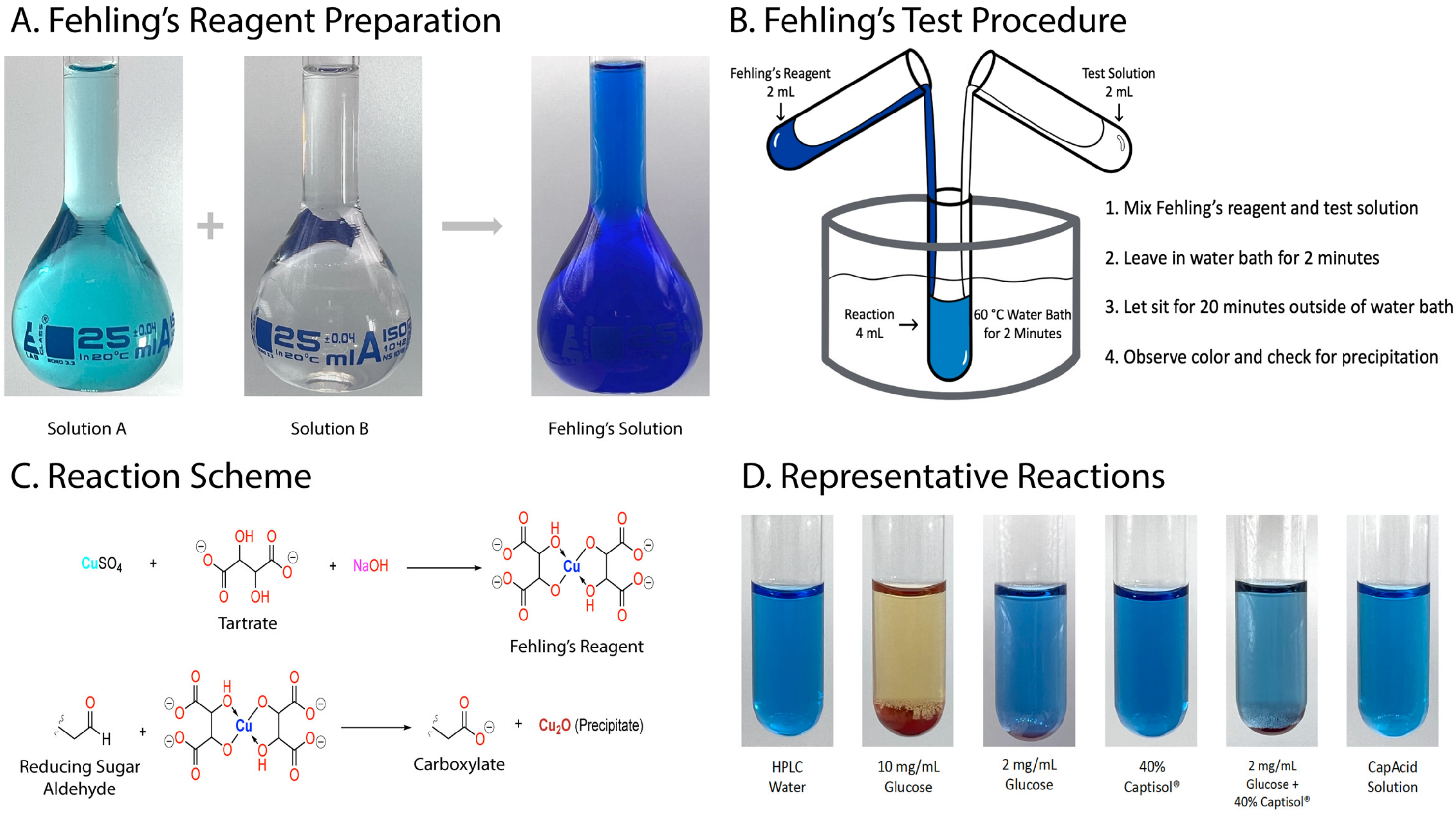

Figure S1. Fehling's Test.

Nuclear Magnetic Resonance Spectra of Ketamine Freebase

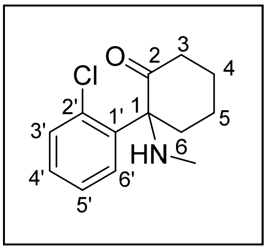

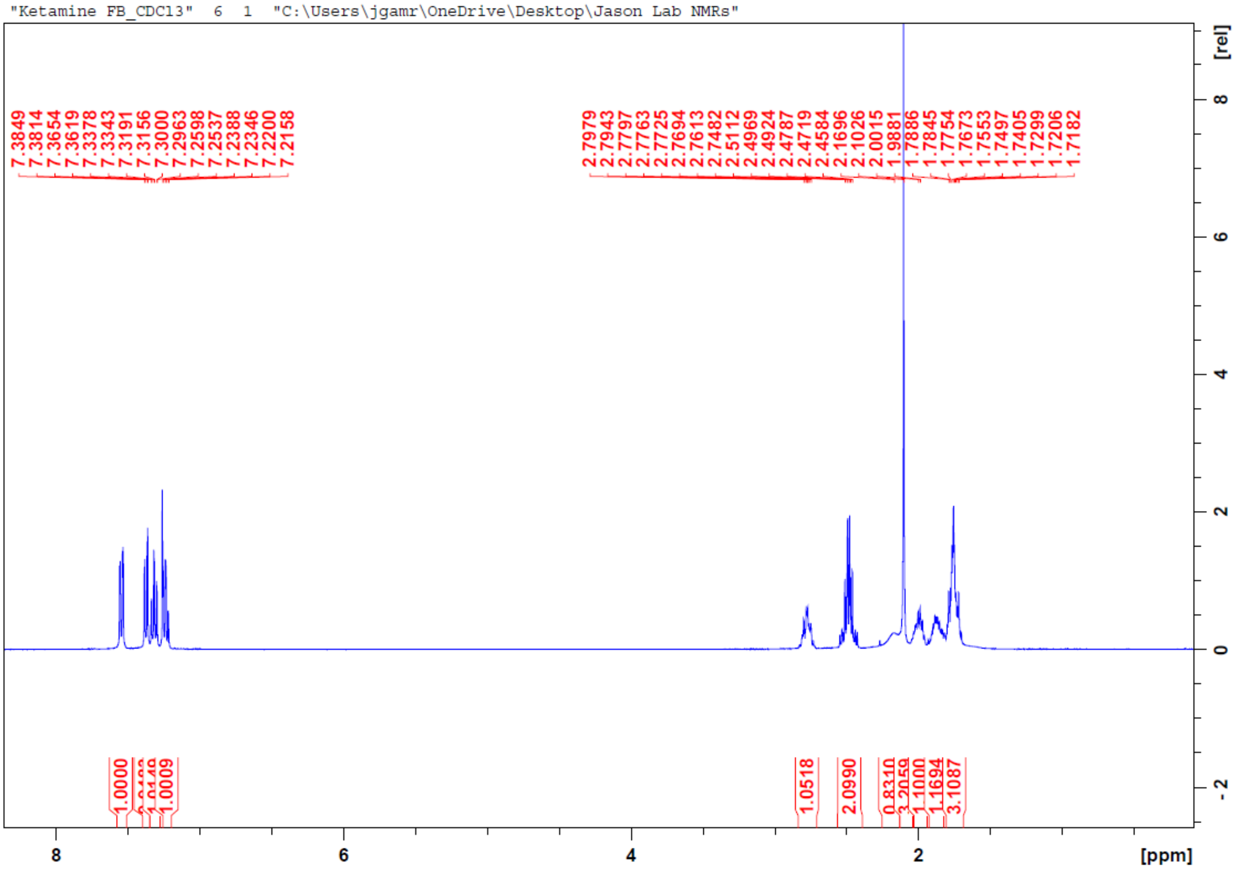

Figure S2a. ^{1}H NMR Spectrum of Ketamine Free Base. Compound dissolved at 20 mg/mL in $CDCl_3$.

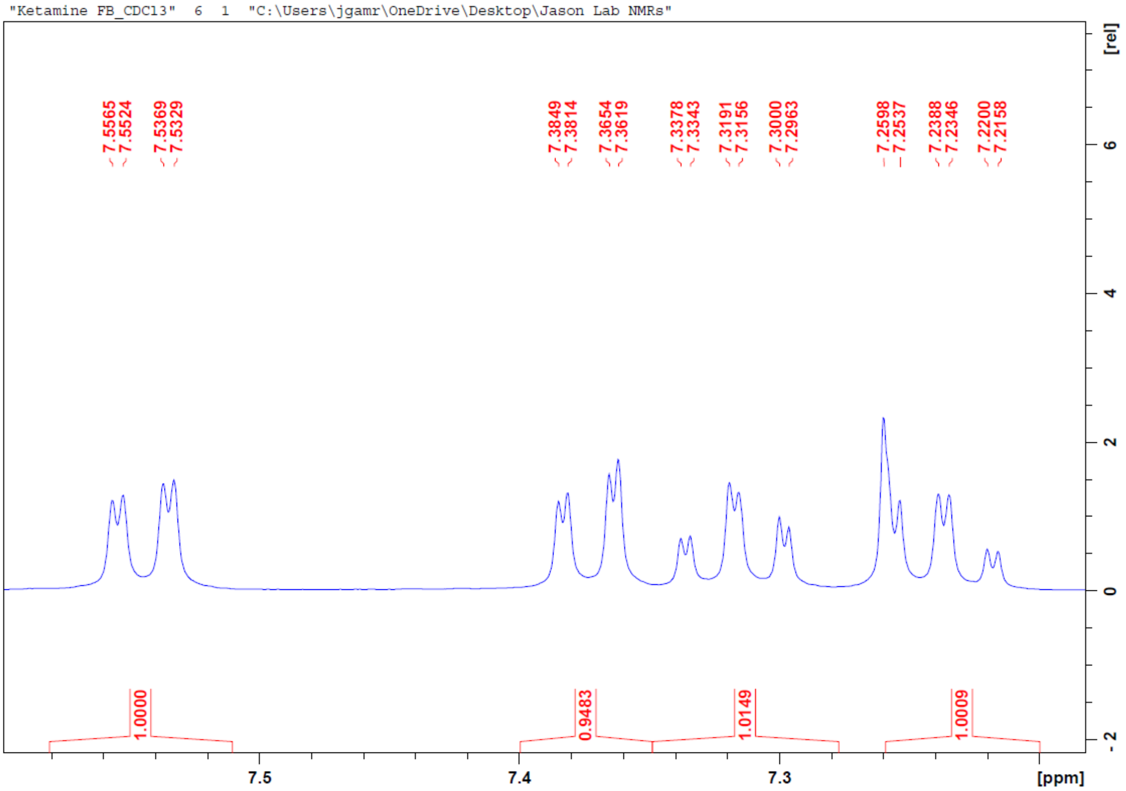

Figure S2b. Aromatic ^{1}H NMR Spectrum of Ketamine Free Base. Compound dissolved at 20 mg/mL in $CDCl_3$.

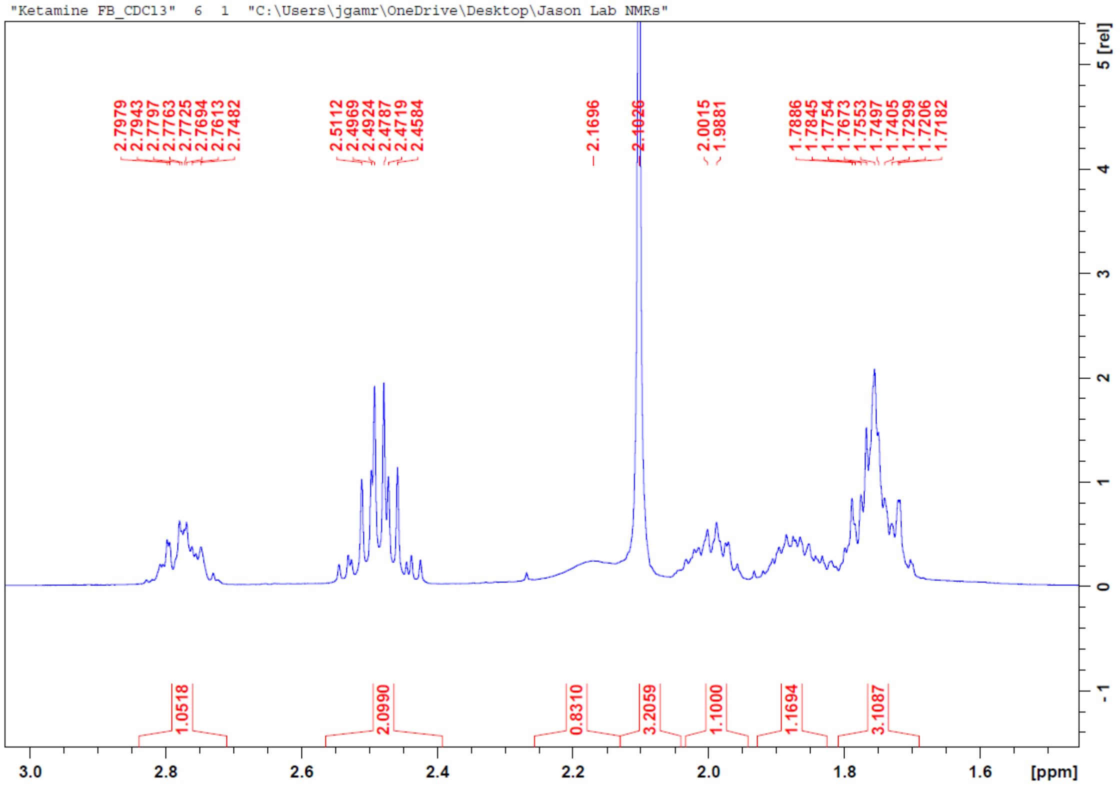

Figure S2c. Aliphatic ^{1}H NMR Spectrum of Ketamine Free Base. Compound dissolved at 20 mg/mL in $CDCl_3$.

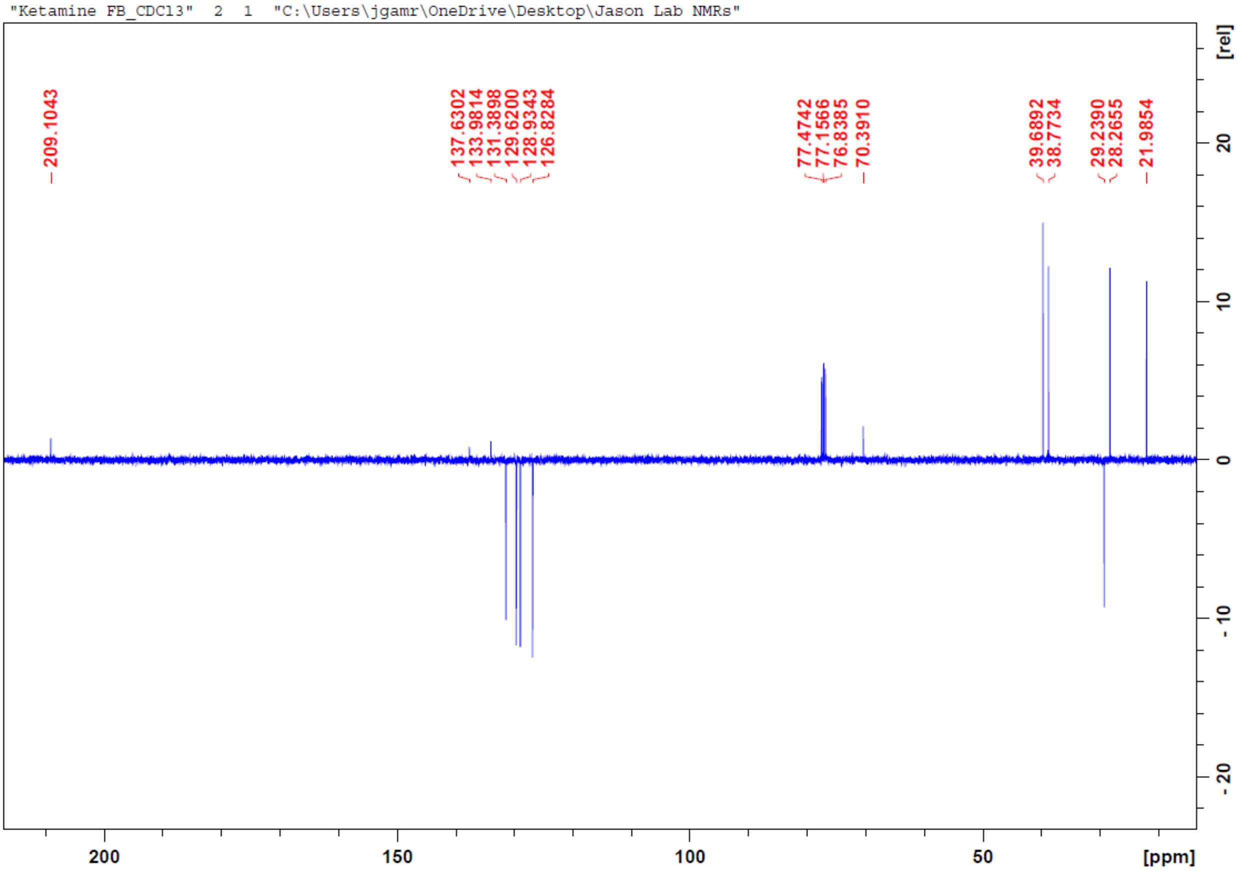

Figure S3. ^{13}C NMR Spectrum of Ketamine Freebase. Compound dissolved at 20 mg/mL in $CDCl_3$.

HPLC analyses

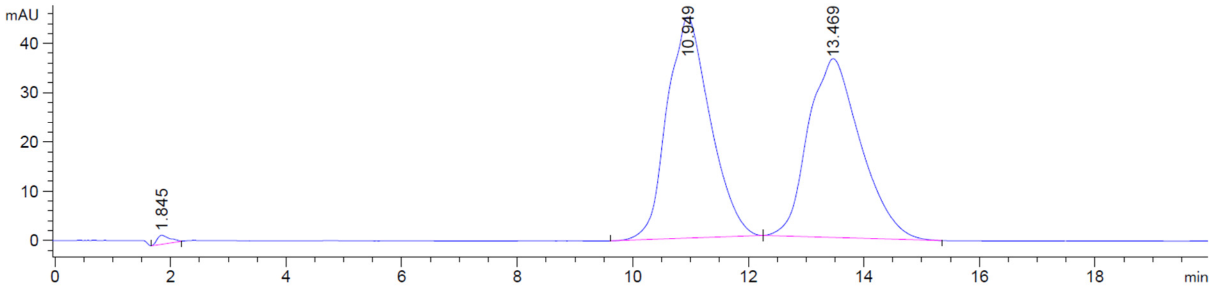

Figure S4. Chiral HPLC of Racemic Ketamine HCl (1 mg/mL).

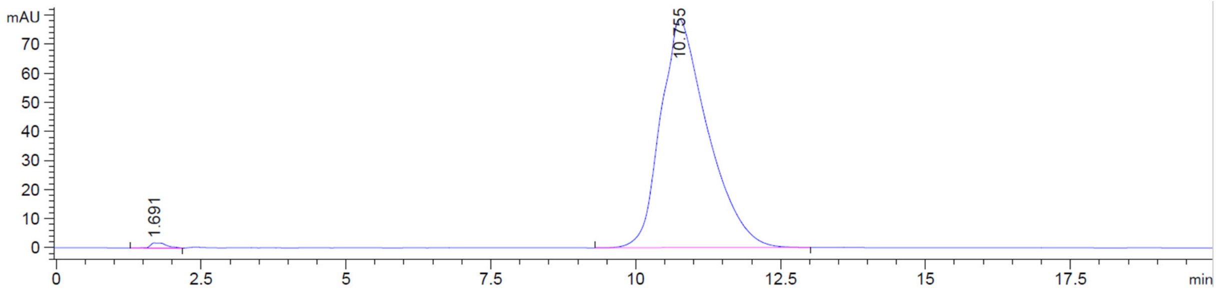

Figure S5. Chiral HPLC of S-Ketamine HCl (1 mg/mL).

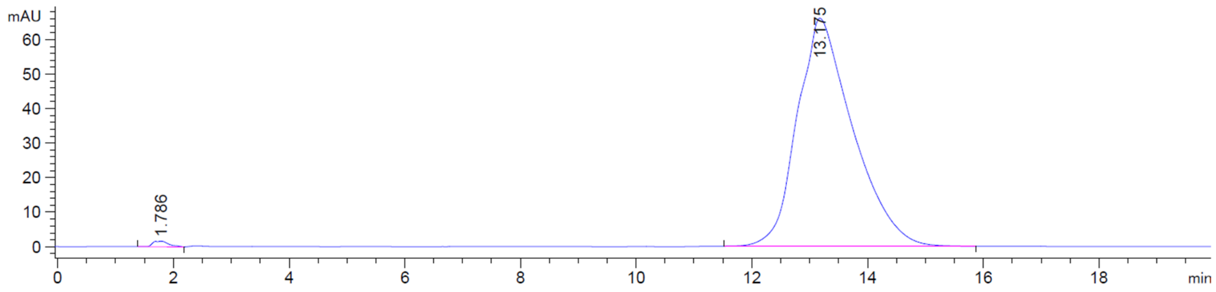

Figure S6. Chiral HPLC of R-Ketamine HCl (1 mg/mL).

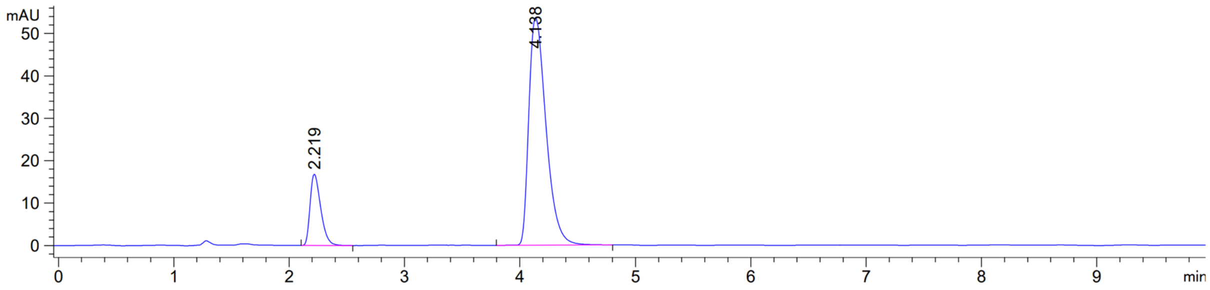

Figure S7. HPLC Trace of BB106; ketamine (4.138 min) with procaine HCl internal standard (2.219 min, 20 µg/mL).

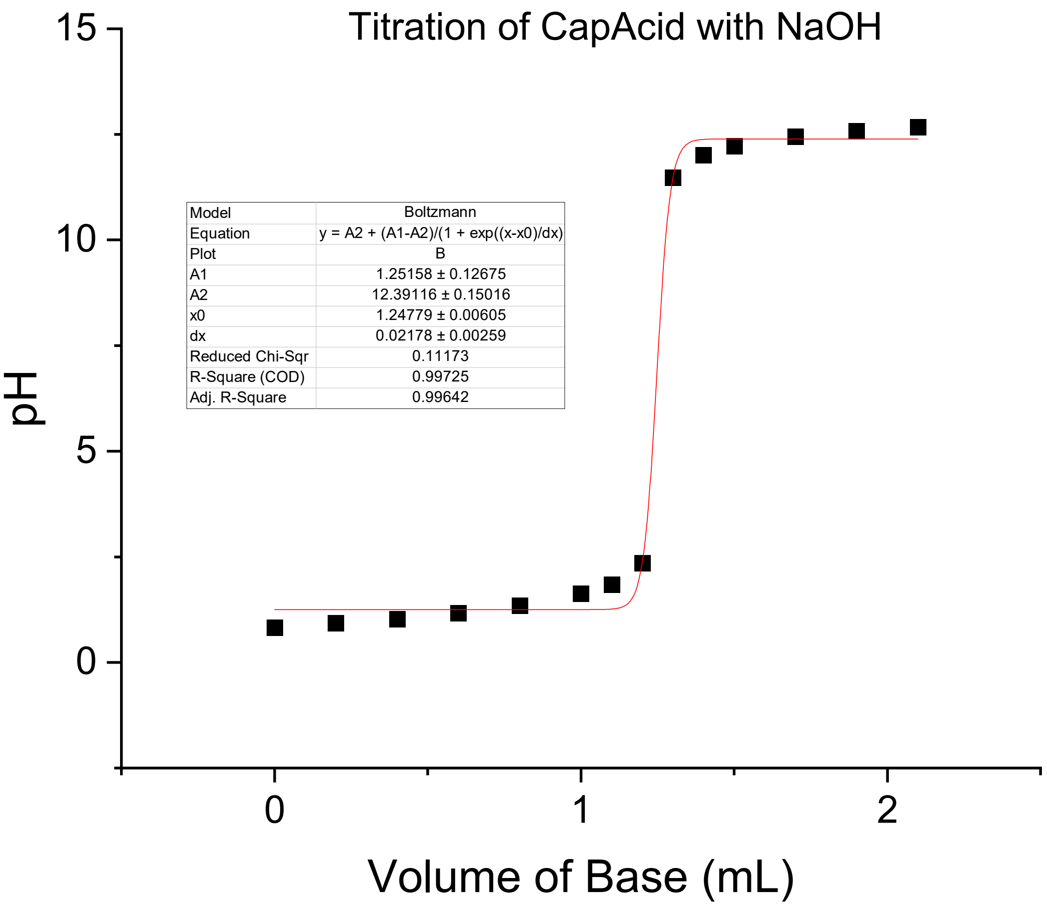

Figure S8. Titration of CapAcid with sodium hydroxide.

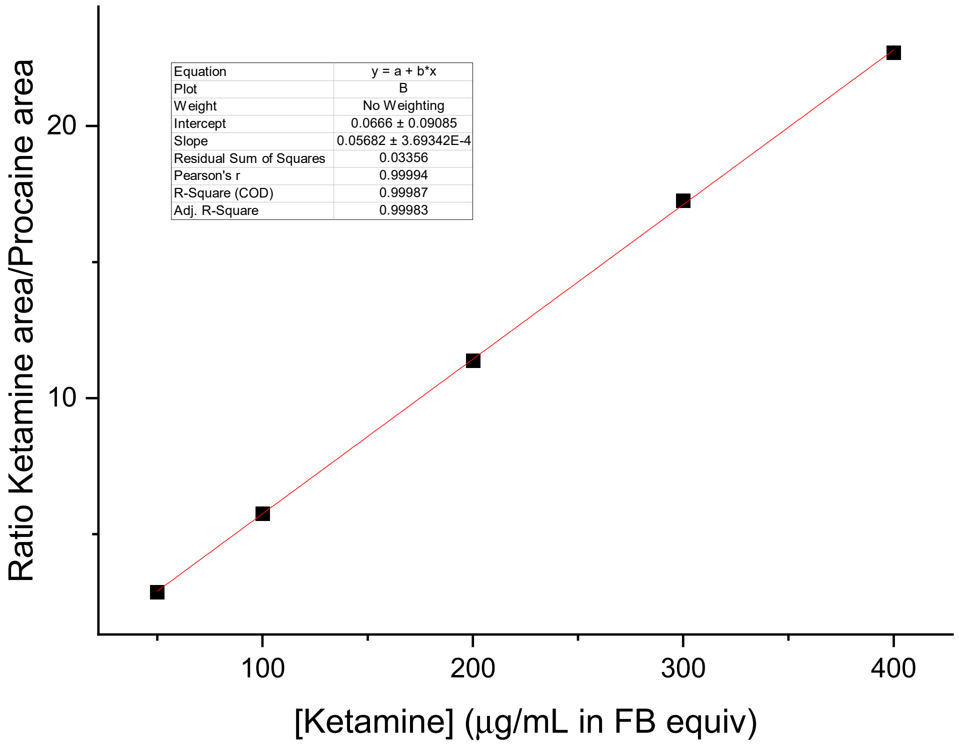

Figure S9. Representative Ketamine HPLC calibration curve.

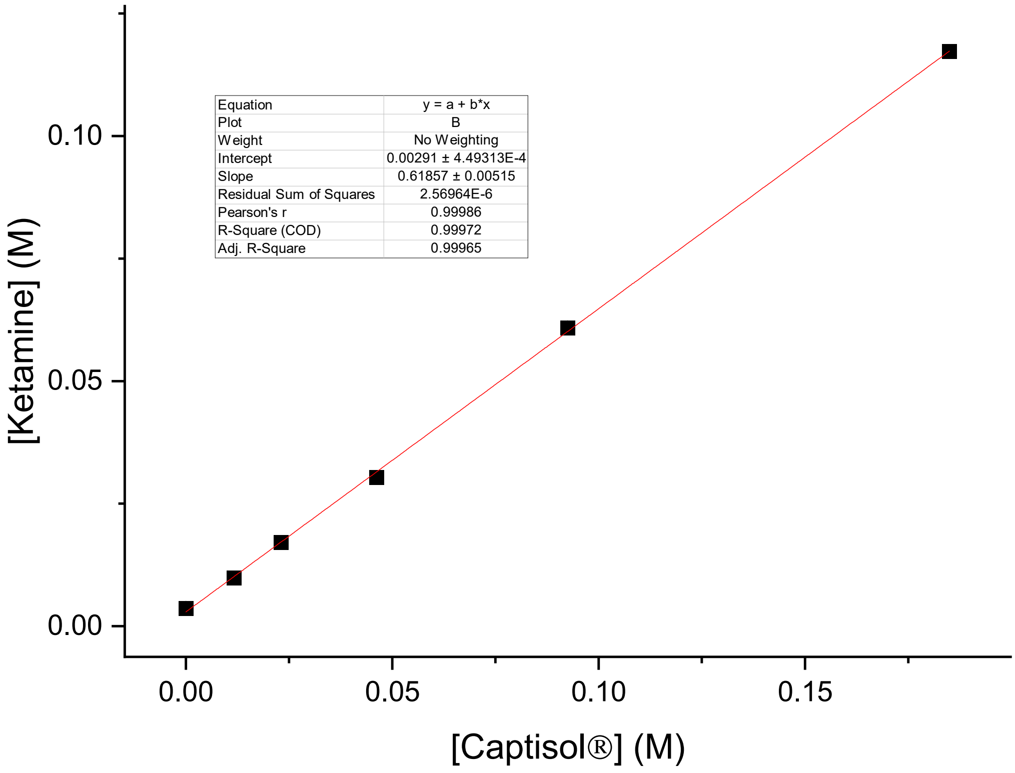

Figure S10. Ketamine Phase Solubility Curve with Captisol®.
